# Supplementary material for: Metabolomics and proteomics analyses reveal the role of the glycerophospholipid metabolism pathway in unexplained recurrent spontaneous abortion
Source: PeerJ. 2025 Apr 30;13:e19317. doi: 10.7717/peerj.19317 (PMC12049100; doi:10.7717/peerj.19317)
Supplement: Supplemental Information 1 [file peerj-13-19317-s001.docx]

**Table S1.** Demonstration of elution gradients for mobile phases in positive and negative ion mode.

| Positive Ion Model | | | | | Negative Ion Mode | | | |
| --- | --- | --- | --- | --- | --- | --- | --- | --- |
| Time (min) | Flow rate (mL/min) | A (%) | B (%) | | Time (min) | Flow rate (mL/min) | A (%) | B (%) |
| 0 | 0.4 | 100 | | 0 | 0 | 0.4 | 100 | 0 |
| 3 | 0.4 | 80 | | 20 | 1.5 | 0.4 | 95 | 5 |
| 4.5 | 0.4 | 65 | | 35 | 2 | 0.4 | 90 | 10 |
| 5 | 0.4 | 0 | | 100 | 4.5 | 0.4 | 70 | 30 |
| 6.3 | 0.4 | 0 | | 100 | 5 | 0.4 | 0 | 100 |
| 6.4 | 0.4 | 100 | | 0 | 6.3 | 0.4 | 0 | 100 |
| 8 | 0.4 | 100 | | 0 | 6.4 | 0.4 | 100 | 0 |
|  |  |  |  |  | 8 | 0.4 | 100 | 0 |
