# Supplementary figures and images for: Metabolomics and proteomics analyses reveal the role of the glycerophospholipid metabolism pathway in unexplained recurrent spontaneous abortion
Source: PeerJ. 2025 Apr 30;13:e19317. doi: 10.7717/peerj.19317 (PMC12049100; doi:10.7717/peerj.19317)

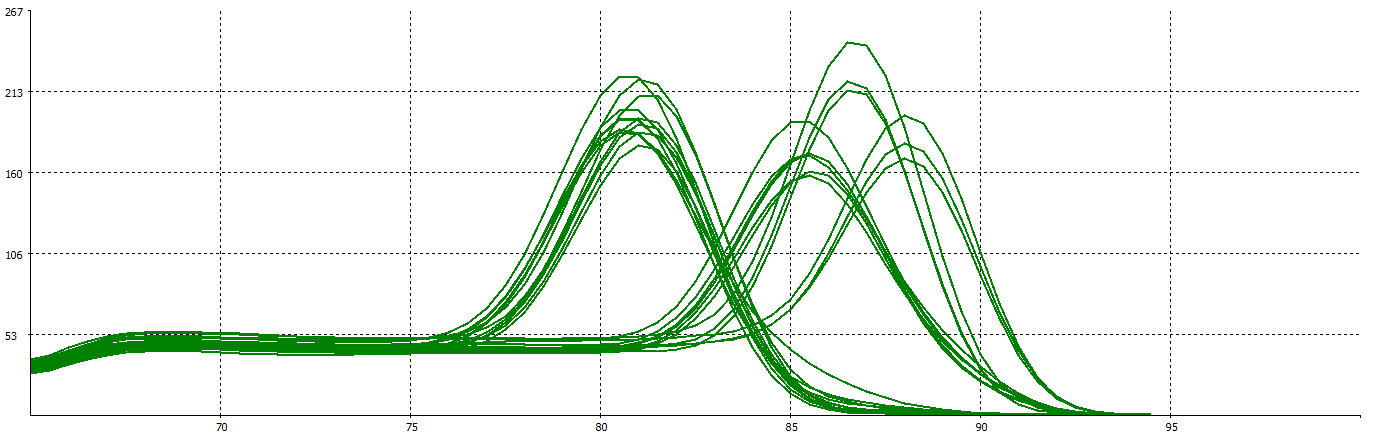

Supplement: Supplemental Information 4 [file peerj-13-19317-s004.zip › PLD1, CHPT1 and PLA2G2A(PCR, cck-8)/derivative curve.jpg]

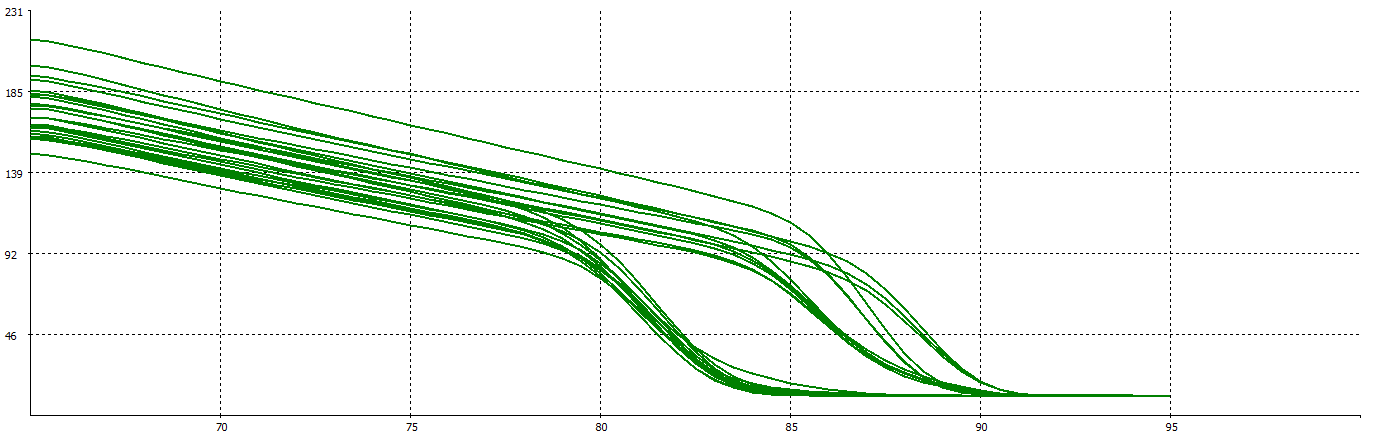

Supplement: Supplemental Information 4 [file peerj-13-19317-s004.zip › PLD1, CHPT1 and PLA2G2A(PCR, cck-8)/dissolution curve.jpg]

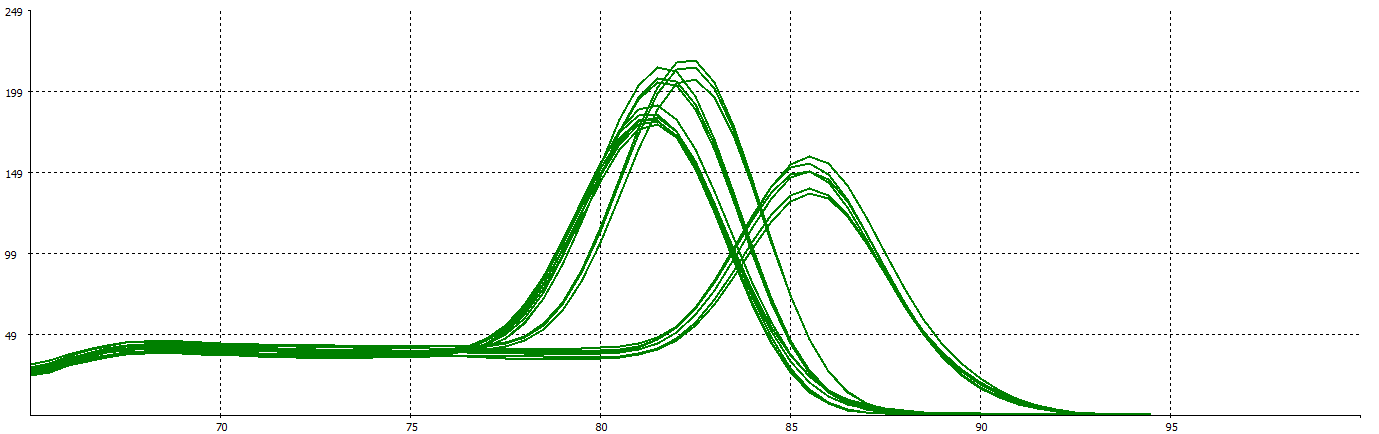

Supplement: Supplemental Information 4 [file peerj-13-19317-s004.zip › PRL and IGFBP1(PCR)/derivative curve.jpg]

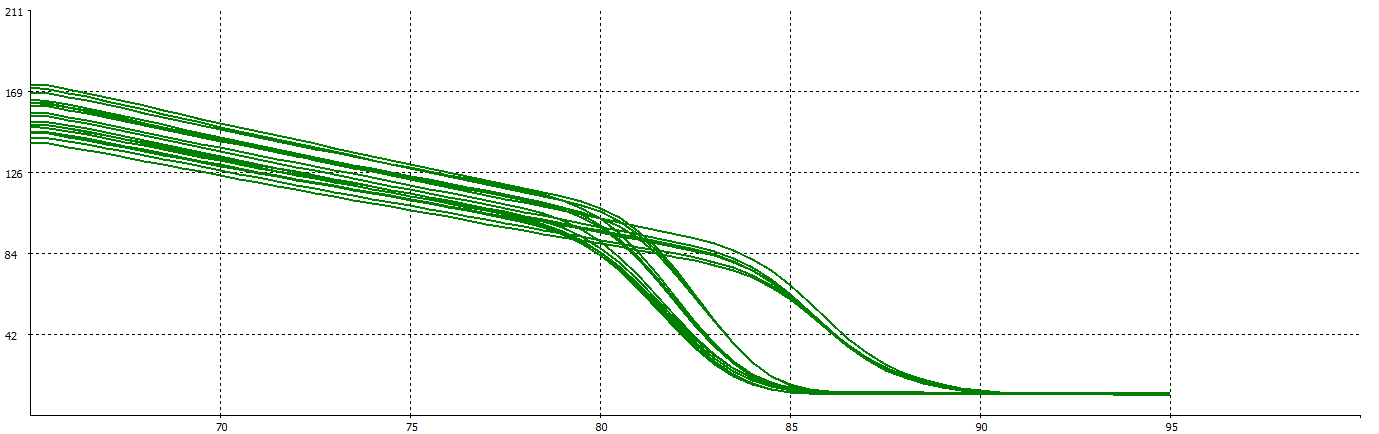

Supplement: Supplemental Information 4 [file peerj-13-19317-s004.zip › PRL and IGFBP1(PCR)/dissolution curve.jpg]
